# A Green Synthetic Approach to Glycerol Trihexanoate from Renewable Feedstocks for Enhanced Plasticization across Diverse Polymer Matrices

**DOI:** 10.1021/acsapm.6c00413

**Published:** 2026-03-29

**Authors:** Luca Lenzi, Laura Martellosio, Marica Bianchi, Andrea Dorigato, Micaela Degli Esposti, Davide Morselli, Paola Fabbri

**Affiliations:** † Civil, Chemical, Environmental and Materials Engineering Department, 9296 Università di Bologna, Via Terracini 28, 40131 Bologna, Italy; ‡ National Interuniversity Consortium of Materials Science and Technology (INSTM), 50121 Firenze, Italy; § Department of Industrial Engineering, Università di Trento, Via Sommarive 9, 38123 Povo, Italy

**Keywords:** biobased plasticizers, polymer additives, glycerol esters, crystallinity, PVC, PHAs

## Abstract

The increasing demand for sustainable polymer additives has raised attention on the development of biobased plasticizers as alternatives to conventional fossil-derived compounds. In this work, glycerol trihexanoate (GTH) was synthesized via a solvent-free reaction between glycerol and hexanoic acid, two renewable, biobased, and nontoxic building blocks. The plasticizing effect of GTH is investigated on a fully amorphous polymer, such as poly­(vinyl chloride) (PVC), and on poly­(3-hydroxybutyrate-*co*-3-hydroxyhexanoate) (PHBH), a biobased semicrystalline polyester. Through a comprehensive multitechnique approach, the thermal, mechanical, morphological, and surface properties of plasticized films are investigated. GTH demonstrated excellent plasticizing efficiency, significantly lowering the glass transition temperature and improving flexibility in both polymers without causing phase separation. Notably, GTH can promote in PHBH an increase of crystallinity and trigger the formation of β-phase crystalline domains, typically associated with enhanced ductility and reduced melting temperatures of the material. Migration tests in water reveal minimal plasticizer leaching (<0.6%), underscoring the remarkable retention of GTH regardless of the polymer. These findings support the compatibility and integration of GTH in different polymer systems and highlight its potentialities as a long-lasting, high-performance, and environmentally friendly plasticizer for both conventional and biodegradable plastic formulations.

## Graphical abstract

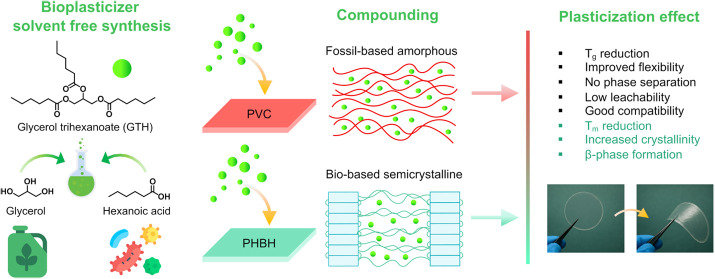

## 1 Introduction

The extensive use of fossil-based plasticizers in polymer formulations has raised significant environmental and health concerns. Conventional plasticizers, particularly phthalates, are known to leach from plastic materials,^1–3^ leading to widespread human exposure through air, food, and water. Such exposure has been linked to endocrine disruption, reproductive toxicity, and carcinogenic effects.^4–6^ Moreover, the production of these plasticizers relies heavily on nonrenewable petrochemical resources, contributing to environmental pollution and carbon emissions. In response to these challenges, the development of biobased plasticizers has gained attention from both the research and industrial worlds. Alternatives such as epoxidized soybean oil, citrates, and sebacates have been explored for their potential to replace traditional plasticizers. However, these alternatives often face limitations, including higher production costs, limited compatibility with various polymers, and lower plasticizing efficiency compared to their fossil-based counterparts.^7–14^ In order to overcome these limitations, in this study, we propose glycerol trihexanoate (GTH) as a biobased plasticizer. Similarly to glycerol-based plasticizers previously developed,^15–17^ also GTH is synthesized through a solvent-free catalyzed esterification of hexanoic acid (HA) with glycerol (GLY). GLY is a well-established byproduct of biodiesel production, typically accounting for around 10 wt % of the total production of the biofuel.^18^ As biodiesel production has expanded globally, glycerol has become an abundant and low-cost resource, and its safe environmental profile and chemical versatility have led to its widespread use as a building block in green chemistry and polymer modification strategies.^19,20^ On the other hand, HA is a promising medium-chain fatty acid widely studied for its applications in the food and agricultural sectors.^21,22^ Due to its molecular structure and biodegradability, HA has attracted increasing interest as a renewable building block in sustainable materials. Recent advances in microbial and biotechnological processes have demonstrated its potential to be produced from renewable feedstocks^23^ and even CO_2_ through microbial electrosynthesis and chain elongation strategies.^24,25^ Importantly, the degradation of glycerol ester-based plasticizers like GTH results in the release of their constituent components, GLY and HA,^26^ which both are recognized for their low toxicity. Glycerol has a high LD_50_ value and is widely used in food, cosmetic, and pharmaceutical applications.^27^ HA is used as a flavoring agent and antimicrobial additive, further supporting the safety profile of GTH.^28,29^ Beyond the synthesis of GTH, this work aimed to systematically evaluate the performance of the biobased plasticizer and to investigate its interaction with two polymeric systems of markedly different nature, such as poly­(vinyl chloride) (PVC) and poly­(3-hydroxybutyrate-*co*-3-hydroxyhexanoate) (PHBH). PVC is a widely used petroleum-based thermoplastic with a long history of plasticizer use, offering a well-established benchmark for evaluating plasticizer efficiency and compatibility.^30^ Its entirely amorphous structure and sensitivity to plasticizer incorporation make it an ideal model system for assessing the fundamental softening behavior and molecular mobility induced by additives.^31^ In contrast, PHBH is a biodegradable semicrystalline polyester belonging to the polyhydroxyalkanoates (PHAs) family, which is attracting increasing attention as a sustainable alternative to conventional petroleum-based polymers. PHBH is a copolymer composed of 3-hydroxybutyrate (3HB) and 3-hydroxyhexanoate (3HH) monomeric units.^32^ The incorporation of the longer and more flexible 3HH units disrupts the regular crystallization pattern of PHB, reducing brittleness and lowering the glass transition temperature, thereby enhancing the material’s flexibility and toughness compared to the PHB homopolymer.^33^ However, despite these improvements, PHBH still exhibits drawbacks typical of PHA-based materials, including limited thermal stability, narrow processing windows, and insufficient flexibility for demanding applications.^34^ To evaluate the potential of GTH as a sustainable plasticizer, its influence on the thermal, mechanical, structural, and surface properties of polymer films was systematically investigated. Differential scanning calorimetry (DSC) was used to study thermal transitions and crystallinity, wide-angle X-ray diffraction (WAXD) provided insight into crystalline structure and possible changes in polymorphism, mechanical behavior of the materials was evaluated through tensile tests, while scanning electron microscopy (SEM) was employed to investigate potential changes in morphology and the compatibility between the polymers and the biobased plasticizer. The impact of GTH on the surface characteristics was examined through dynamic contact angle measurements, while migration tests offered information about the physical stability of the plasticized formulations.

This study aims to provide a simple and effective green approach to synthesize an innovative bioplasticizer starting from sustainable-derived feedstocks and thus proposes a valuable alternative for overcoming the limitations of the commercial plasticizers. Moreover, a detailed understanding of the GTH/polymer interactions and the related structure–property relationship is herein proposed. By exploration of these interactions, this work also contributes to the growing knowledge needed for the rational design of safer and more sustainable plasticizer systems capable of reducing the environmental footprint of plastic materials.

## 2 Experimental Section

### 2.1 Materials

Hexanoic acid (HA, ≥99%), glycerol (GLY, ≥99%), hexane (Hex ≥ 99%), ethyl acetate (EtOAc ≥ 99%), ethanol (EtOH, ≥99,8%), sodium sulfate (Na_2_SO_4_, anhydrous, ≥99%), sodium carbonate (Na_2_CO_3_, ≥99.5%), and sodium chloride (NaCl) were all purchased from Sigma-Aldrich. Additionally, water (HPLC grade), p-toluenesulfonic acid monohydrate (PTSA, 98.5%, Alfa Aesar), and deuterated chloroform (CDCl_3_, 99.8 atom % D, tetramethylsilane (TMS) content 0.03% v/v, WVR Chemicals). Thin-layer chromatography (TLC) precoated aluminum-backed plates were used (Merck Kieselgel 60 F254) and visualized using a solution of potassium permanganate (KMnO_4_, 0.06 M). The above-mentioned reagents and solvents were used as received without further purification. PVC (industrial grade, *M*_w_ = 150,300) was provided by Resilia Srl (Italy) and PHBH (IamNATURE B6 A13, *M*_w_ = 610,000; 3HH content = 6 mol %) was supplied by MAIP Srl (Italy). Before their use, both polymers were carefully purified in order to remove residual additives and contaminants. Briefly, PVC was dissolved in tetrahydrofuran (THF) at a concentration of 0.67 mg·mL^–1^, while PHBH was solubilized in chloroform (CHCl_3_) under the same conditions. The resulting solutions were filtered under vacuum using Celite as a filtering medium and precipitated in an excess of cold methanol.^35^ The purified polymers were collected, dried, and stored under a vacuum until use.

### 2.2 Synthesis of Glycerol Trihexanoate (GTH)

GTH was synthesized via solvent-free esterification of GLY and HA in a 1:10 molar ratio. The reagents were placed in a 250 mL round-bottom flask equipped with a magnetic stirrer and heated in a silicon oil bath at 100 °C. After stabilization, PTSA was added at 3% with respect to GLY moles, and the reaction proceeded for 24 h under continuous stirring, keeping the flask open in order to allow water removal. When the reaction ended, the mixture was neutralized with saturated solution of Na_2_CO_3_, and the product was separated through liquid–liquid extraction. The aqueous phase was washed twice with Hex, and the collected organic phase was subsequently washed once with Na_2_CO_3_, followed by a 1:1 H_2_O/EtOH solution and finally with a saturated NaCl solution (brine). The organic phase was dried over anhydrous Na_2_SO_4_, and the solvent was removed under reduced pressure by using a rotary evaporator. The purified yellowish liquid was dried overnight in an oven at 40 °C.

### 2.3 GTH Synthesis Monitoring

The progression of the synthesis of glycerol trihexanoate (GTH) was monitored over time using Fourier transform infrared spectroscopy (FTIR). Specifically, spectra were recorded using a PerkinElmer Spectrum Two spectrometer equipped with a diamond attenuated total reflection (ATR) crystal. The spectral window was set from 4000 to 400 cm^–1^ with 16 scans per sample. Finally, the spectrum of purified GTH was recorded. Spectral data were analyzed with Spectrum 10 software (PerkinElmer). In parallel, the reaction progress and product purity were monitored by thin-layer chromatography (TLC) using silica gel plates and Hex/EtOAc (6:4 volume ratio) as the mobile phase. The plates were visualized using a KMnO_4_ staining solution.

### 2.4 Nuclear Magnetic Resonance (NMR) Spectroscopy

After the presence of only the desired product was ensured, the chemical structure of GTH was validated by ^1^H and ^13^C nuclear magnetic resonance (NMR) spectroscopy. Spectra were acquired at room temperature using a Bruker Avance 400 MHz spectrometer with deuterated chloroform (CDCl_3_) as the solvent and tetramethylsilane (TMS) as an internal reference. To provide further structural confirmation, two-dimensional (2D) NMR techniques were employed, including correlation spectroscopy (COSY), heteronuclear single quantum coherence (HSQC), and heteronuclear multiple bond correlation (HMBC). The spectra were processed with TopSpin 4.5.0 (Bruker). Detailed assignments of the ^1^H NMR and ^13^C NMR spectra are reported in the Supporting Information using the following abbreviations to indicate the multiplicity in NMR spectra: s, singlet; d, doublet; t, triplet; m, multiplet.

### 2.5 Polymeric Films Preparation

Neat and plasticized polymeric films were prepared by the solvent casting method. Specifically, PVC and PHBH were separately dissolved in THF and CHCl_3_, respectively, at a concentration of 50 mg·mL^–1^. GTH was then added to the polymer solutions at 0, 10, 20, and 40 ppm resin (phr) under stirring until complete homogenization. The solutions were then poured into Petri dishes and left to dry at room temperature under a fume hood for 24 h. The obtained films were further dried in a vacuum oven at 50 °C for an additional 24 h to remove residual solvents. The final thickness of the films was approximately 100 μm, and the incorporation of the plasticizer did not significantly altered their appearance, as shown in the images reported in Figure S1. Samples were nominated as nPVC and nPHBH for the neat polymers, while the plasticized formulations were labeled as PVC*x*GTH and PHBH*x*GTH, where *x* indicates the GTH content in phr (10, 20, or 40).

### 2.6 Differential Scanning Calorimetry (DSC)

Thermal properties were analyzed using a TA Instruments DSC Q100 calorimeter to assess the influence of GTH on the thermal behavior of PVC and PHBH. The thermograms were analyzed using TA Universal Analysis 2000 software (TA Instruments). Samples (∼3 mg each) were initially cooled to −30 °C and then heated to 195 °C at a rate of 20 °C·min^–1^ under a nitrogen atmosphere to eliminate thermal history. Following this step, the samples were cooled at 10 °C·min^–1^ to −90 °C and subjected to a second heating scan at 20 °C·min^–1^ to determine the glass transition temperature (*T*_g_) for both PVC and PHBH formulations. This faster heating rate was selected to enhance the detectability of *T*_g_, which otherwise may appear less evident at slower heating rates. Since PHBH is semicrystalline, an additional heating cycle at 10 °C·min^–1^ up to 195 °C was conducted after cooling at 10 °C·min^–1^ to −90 °C. This slower heating cycle allowed a more careful evaluation of the cold crystallization temperature (*T*_cc_), cold crystallization enthalpy (Δ*H*_cc_), melting temperature (*T*_m_), and melting enthalpy (Δ*H*_m_). The latter was subsequently used in eq 1 to calculate the crystalline percentage of PHBH in each formulation

1 $$X_{C}\;\left(\%\right)={[(\Delta H}_{m}-{\Delta H}_{cc})/{(W}_{Pol}\times {\Delta H}_{m}^{0})]\times 100$$

where *W*_Pol_ represents the fraction of polymer in the compound and Δ*H°*_m_ is the standard melting enthalpy for PHBH (146 J·g^–1^).^36^

### 2.7 Wide-Angle X-ray Diffraction (WAXD)

The crystalline structure and phase composition of PHBH neat and plasticized films were analyzed by X-ray diffraction (XRD) measurements performed on a PANalytical X’Pert PRO powder diffractometer equipped with a Cu Kα ceramic X-ray tube (λ = 1.5418 Å) operating at 40 kV and 30 mA and with a fast X’Celerator detector. The characterizations were performed in air and at room temperature, in parallel-beam geometry and symmetric reflection mode (2θ range 5–55°, step time of 564 s, and step size of 0.1°/2θ), repeating the measurement four times to reduce the noise. Prior to analysis, semicrystalline films were conditioned in a hydraulic press for 30 min using flat heated platens at 100 °C and then cooled to room temperature to provide an equal treatment to all samples and thus make them comparable. This treatment allowed the crystallizable macromolecular segments to reorganize into ordered domains, thereby promoting the formation of highly crystalline samples. To enhance the distinction of overlapping diffraction peaks, spectral deconvolution was applied, and the resulting fitted XRD patterns are provided in the Supporting Information (Figure S2). Data were analyzed and processed using PANalytical HighScore 5.1a software. In order to observe the variations of the α and β-phase with respect of the increasing content of the plasticizer, the ratios of individual peak areas (*A*_phase(*x*)_^*^), for each characteristic crystallographic planes for the two phases ([020] and [011] for α-phase and [110] for β-phase) were calculated by comparing the normalized area of a given peak in samples containing GTH (*A*_phase(*x*),plast_) to that of the corresponding peak in the neat polymer (*A*_phase(*x*),neat_), as expressed in eq 2

2 $$\begin{array}{ll} A_{\mathrm{phase}\left(x\right)}^{*}=A_{\mathrm{phas}e\left(x\right),\mathrm{plast}}/A_{\mathrm{phase}\left(x\right),\mathrm{neat}}; & \\ \mathrm{phase}=\alpha ,\beta ; & x=\left[020\right],\left[110\right],\left[011\right] \end{array}$$

Moreover, to emphasize the variation of the β-phase (Figure S2), of PHBH, by changing the content of GTH, *A*_β[011]_^*^ was related to *A*_α(x)_^*^ through eq 3

3 $$\beta_{011}^{*}/\alpha_{x}^{*}=A_{\beta \left(011\right)}^{*}/A_{\alpha \left(x\right)}^{*};,x=\left[020\right];\left[011\right]$$

### 2.8 Tensile Testing

The mechanical properties of plasticized films were assessed via tensile tests using an INSTRON 5966 universal testing machine equipped with a 10 kN load cell. Rectangular specimens (10 mm width and 70 mm length) were cut from the films, and the gauge length was set to 20 mm. Tensile tests were performed at a crosshead speed of 4 mm·min^–1^. Young’s modulus (*E*) and elongation at break (ε_break_) were determined for each formulation, with three replicates.

### 2.9 Scanning Electron Microscopy (SEM)

The morphology of the samples and the possible phase separation between the polymers and the plasticizer were examined by using field emission scanning electron microscopy (FE-SEM). Cross sections of the polymeric films were prepared by cryo-fracturing in liquid nitrogen, followed by a gold coating (10 nm thickness) via electrodeposition to ensure electrical conductivity. The observations were carried out using a Nova NanoSEM 450 electron microscope (FEI Company, Bruker Corporation) operated at an accelerating voltage of 10 kV.

### 2.10 Migration Tests

Leaching of GTH was assessed by performing a migration test. Samples with a surface area of 1 cm^2^ were placed in sealed vessels containing 50 mL of Milli-Q water and kept under gentle stirring at room temperature for 24 h. The weight loss (%) was calculated using eq 4

4 $$weight\;loss\;\left(\%\right)={[(W}_{i}-W_{f})/W_{i}]\times 100$$

where *W*_i_ is the initial weight of the sample before immersion and *W*_f_ is the final weight after extraction tests and subsequent drying in a thermostatic oven at 50 °C for 24 h.

### 2.11 Contact Angle and Surface Wettability Investigations

The surface wettability of the plasticized PVC and PHBH films was evaluated through dynamic contact angle measurements carried out with a Force Tensiometer (Sigma 700/701, Biolin Scientific) operating with the Wilhelmy plate method using water as the probe liquid. Advancing (θ_a_) and receding (θ_r_) contact angles were measured for each formulation, and the equilibrium contact angle (θ_0_) was calculated through eq 5, using an energy-based approach that accounts for the geometry of the triple-phase contact line^37^

5 $$\cos\left(\theta_{0}\right)=\left[\Gamma_{A}\cdot \cos\left(\theta_{a}\right)+\Gamma_{R}\cdot \cos\left(\theta_{r}\right)\right]/\left[\Gamma_{A}+\Gamma_{R}\right]$$

where Γ_A_ and Γ_R_ are geometric weighting factors calculated from eq 6

6 $$\Gamma_{X}={\left[\left(\sin^{3}\theta_{X}\right)/\left(2-3\cdot \cos\theta_{X}+\cos^{3}\theta_{X}\right)\right]}^{1/3};,X=A,R$$

θ_a_ and θ_r_ were measured through five repetitions for each sample, with the first and last measurements excluded from the analysis to minimize the influence of surface conditioning and end effects.

## 3 Results and Discussion

### 3.1 GTH Synthesis and Characterization

Similarly to other glycerol-based plasticizers,^16,17^ GTH was synthesized via a solvent-free esterification, in which GLY and HA were reacted with an excess of the acid, using PTSA as the catalyst. (Figure 1A). The reaction was carried out at 100 °C for 24 h, keeping the flask open, allowing water removal and thus pushing the reaction toward the desired product, and the progress of the esterification was monitored by FTIR analysis. The spectra of the pure reagents are shown in Figure S3A, while Figure 1B displays the full spectra of the reaction mixture collected at different time intervals, as well as that of the purified product. The progressive disappearance of the broad hydroxyl stretching band between 3000 and 3500 cm^–1^, attributed to unreacted GLY, is clearly visible in the inset highlighting this spectral region (Figure S3B), confirming the gradual consumption of GLY over the time of the reaction. At the same time, the increasing intensity of the ester C = O stretching band at 1739 cm^–1^ (Figure 1C) provided evidence of the successful formation of the triester product. The peak observed at 1706 cm^–1^, corresponding to the carboxylic acid groups of excess HA (Figure 1C), remained detectable after 24 h of reaction as expected, due to the large molar excess of HA used. However, the characteristic HA signals completely disappeared after the workup procedure, demonstrating the effectiveness of the applied purification protocol in removing unreacted HA and isolating the final product. Furthermore, TLC was employed in parallel to detect the formation of any potential secondary products. As shown in Figure S4, the chromatographic analysis displayed a single well-defined spot after the purification process, confirming the purification of GTH from possible secondary products. The combination of the adopted reaction conditions and purification steps allowed for the isolation of a pale yellow, odorless liquid with a molar yield of 85%, significantly higher than the yields typically reported for the conventional synthesis of GTH employing PTSA as catalyst.^38^ The structure of GTH was further confirmed through detailed NMR analysis, including ^1^H, ^13^C, and 2D NMR spectroscopy. The ^1^H NMR spectrum, shown in Figure 1D, displayed characteristic signals for the glycerol backbone where the multiplet at 5.27 ppm was assigned to the central proton of glycerol, while two double doublets at 4.29 and 4.15 ppm corresponded to the diastereotopic methylene protons adjacent to the ester groups (highlighted in Figure S5A). The presence of ester-linked alkyl chains was confirmed by peaks between 2.33 and 0.89 ppm, which were attributed to the methylene and methyl groups of the hexanoate moieties. The ^13^C NMR spectrum presented in Figure S5B further supported these findings, with clear resonances at 173.1 and 172.9 ppm indicating the presence of ester carbonyls. The signals at 69.86 and 62.1 ppm were assigned to the glycerol moiety, while the aliphatic chain carbons were identified between 34 and 14 ppm. To gain deeper structural insights, 2D NMR experiments were performed. The COSY spectrum (reported in Figure S6A) revealed scalar coupling between adjacent protons, confirming connectivity within the glycerol backbone and hexanoate chains. HSQC analysis shown in Figure S6B allowed for direct correlation between proton and carbon chemical shifts, reinforcing the assignments made in ^1^H and ^13^C NMR spectra. Finally, HMBC provided long-range correlations between hydrogen and carbon nuclei separated by two or three bonds, offering further confirmation of the complete esterification reaction (Figure S6C).

**Figure 1:**
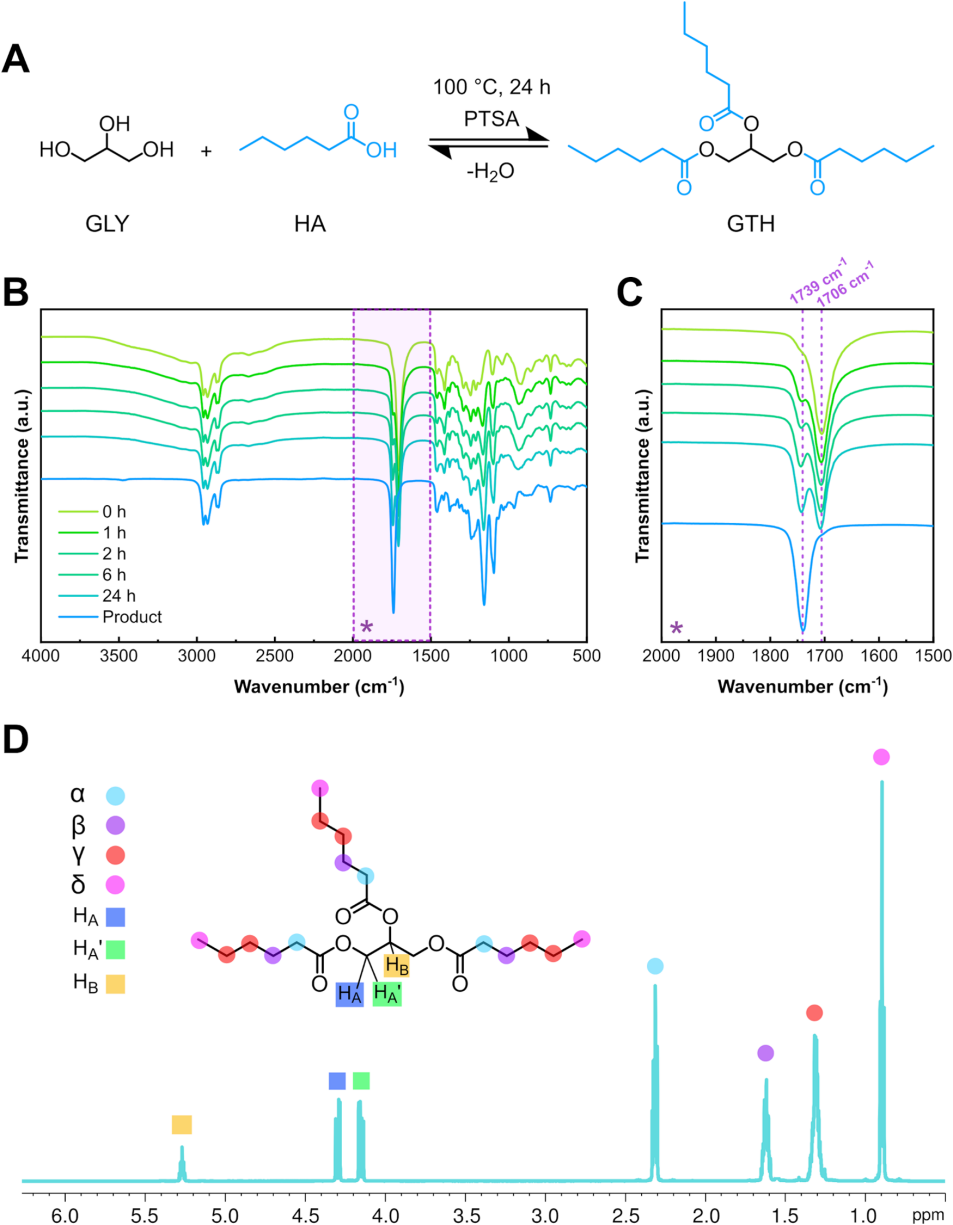
(A) Reaction schematic of the solvent-free esterification of glycerol and hexanoic acid to synthesize GTH. (B) FTIR spectra of reaction intermediates over time, showing the decrease of hydroxyl groups and formation of ester functionalities. (C) Magnified region of FTIR spectra highlighting the C = O signals for the formed ester (1739 cm^–1^) and unreacted acid (1706 cm^–1^). (D) ^1^H NMR spectrum of the purified GTH and related peak assignments.

### 3.2 Thermal Properties

After the synthesis and characterization of GTH, the plasticizer was incorporated into PVC and PHBH via a solvent casting technique, in order to investigate its plasticizing effect on two different polymeric matrices. First, the plasticization of the matrices was assessed by monitoring variations in thermal properties through DSC analysis, and the results are reported in Table S1. Full thermograms of the first heating cycle of PVC (20 °C·min^–1^) used for the extrapolation of the *T*_g_ are reported in Figure S7A, while for PHBH formulations full thermograms of first (20 °C·min^–1^, used to extrapolate the *T*_g_) and second heating cycle (10 °C·min^–1^, used for the extrapolation of the *T*_cc_, Δ*H*_cc_, *T*_m_ and Δ*H*_m_) are reported in Figure S7B and S7C, respectively. The DSC thermograms of the PVC-based formulations (Figure 2A) revealed a progressive reduction in *T*_g_ with increasing GTH content. As shown in Figure 2B, the *T*_g_ of neat PVC decreased from 53 °C to 37, 9, and −3 °C for PVC10GTH, PVC20GTH, and PVC40GTH, respectively, indicating effective plasticization. A similar trend was observed for PHBH, as illustrated in the DSC curves in Figure 2C. The incorporation of GTH led to a marked decrease in *T*_g_, confirming its ability to enhance the chain mobility within the semicrystalline matrix. As reported in Figure 2D, the *T*_g_ of neat PHBH (3 °C) decreased to −14 and −26 °C with 10 and 20 phr of GTH, respectively. Although the observed reduction in *T*_g_ was comparable or even superior to that achieved with other biobased plasticizers,^39^ further increasing the GTH content to 40 phr did not result in a substantial additional *T*_g_ reduction, suggesting that the biobased additive reached a plasticization limit between 20 and 40 phr in this polymer. In addition to the *T*_g_ drop, DSC analysis also provided insights with respect to the cold crystallization and melting characteristics of the PHBH-based formulations. The thermograms collected during the second heating scan (Figure 2E) revealed that all samples underwent so-called cold crystallization, a feature typical of semicrystalline polymers with low initial crystallinity. The neat PHBH sample exhibited a cold crystallization peak at 44 °C. Upon incorporation of GTH, a gradual decrease in *T*_*cc*_ was observed. In fact, the temperature dropped to 33 °C, 19 °C, and 20 °C for PHBH10GTH, PHBH20GTH, and PHBH40GTH respectively, as reported in Figure 2F. This reduction in *T*_cc_ could be attributed to enhanced chain mobility induced by the plasticizer, which facilitated the ordering of macromolecular chains at lower temperatures and consequently promoted the cold crystallization phenomena. The evolution of the melting behavior is illustrated in the thermograms in Figure 2G. Neat PHBH displayed a melting peak at 148 °C, which progressively shifted to lower temperatures upon GTH addition. Specifically, *T*_m_ decreased to 143 °C, 142 °C, and 140 °C for the 10, 20, and 40 phr formulations, respectively (Figure 2H). This trend reflects changes in crystalline order due to the incorporation of GTH, which likely interfered with the regular packing of polymer chains, yielding less thermally stable crystalline domains. The *X*_c_, calculated using eq 1 from the Δ*H*_m_ values (reported in Table S1), provided further insight into the impact of GTH on the semicrystalline morphology of PHBH. As shown in Figure 2H, neat PHBH exhibited a low crystallinity of only 4%, consistent with its limited ability to crystallize due to the presence of 3HH units. Interestingly, the addition of GTH significantly promoted the *X*_c_, which increased to 37%, 39%, and 41% for PHBH10GTH, PHBH20GTH, and PHBH40GTH, respectively. This increase in crystallinity demonstrated that the presence of GTH effectively enhanced the chain mobility, allowing the polymer chains to organize into crystalline domains. The concurrent decrease in *T*_cc_ and increase in *X*_c_ support the hypothesis that GTH facilitates molecular rearrangement and affects crystallization kinetics, possibly by acting as a lubricant at the molecular level.^40^

**Figure 2:**
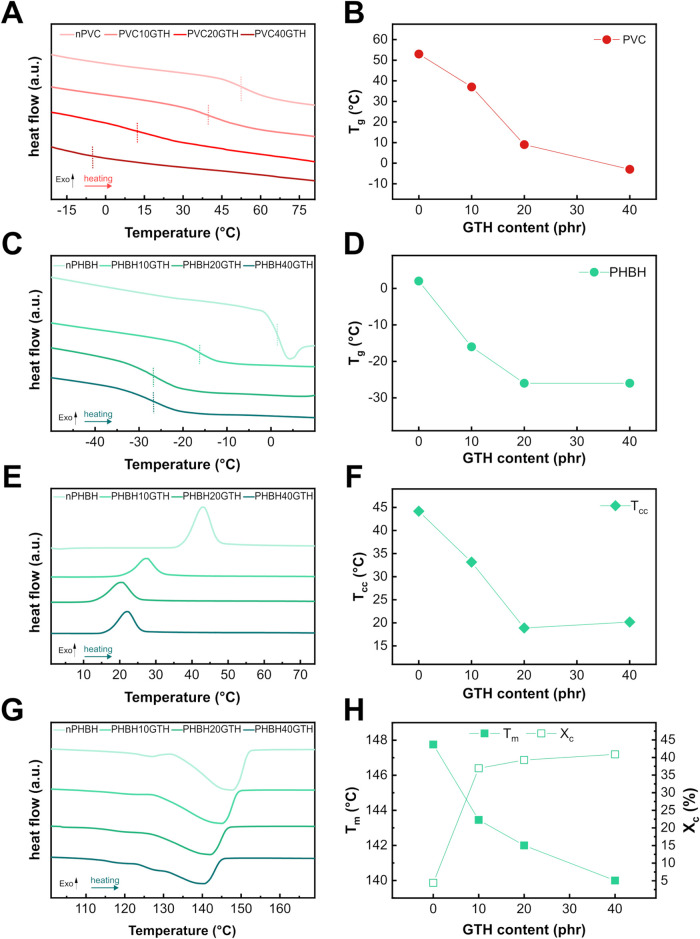
(A) First heating scan (20 °C·min^–1^) DSC thermograms of PVC formulations with increasing GTH content. (B) Glass transition temperature (*T*_g_) of PVC-based samples as a function of GTH content. (C) First heating (20 °C·min^–1^) DSC thermograms of PHBH formulations. (D) Glass transition temperature (*T*_g_) of PHBH-based samples as a function of GTH content. (E) Second heating scan (10 °C·min^–1^) DSC thermograms of PHBH formulations to highlight the cold crystallization peaks. (F) Cold crystallization temperature (*T*_cc_) of PHBH compounds. (G) DSC thermograms of the second heating scan (10 °C·min^–1^) of PHBH compounds, highlighting the melting peaks. (H) Melting temperature (*T*_*m*_) and crystallinity degree (*X*_c_) of PHBH-based samples calculated from DSC curves using eq 1.

### 3.3 Effect of GTH on the PHBH Crystalline Structure

The crystalline fraction of PHBH plays a crucial role in determining its properties, such as mechanical strength and processability. As a semicrystalline biopolymer, its performance in practical applications is strongly influenced not only by the degree of crystallinity but also by the specific nature of its crystalline domains. Modifications of the crystalline structure, such as variations in the crystal size, polymorphism, or packing density, can directly influence the final properties of PHBH-based materials. This is particularly relevant when additives such as plasticizers are introduced, as they may induce significant rearrangements in the crystalline microstructure.^39^ PHBH, like its homopolymer PHB and other PHAs,^41^ crystallizes in the orthorhombic α-phase structure.^42^ In this lattice, the 3-hydroxybutyrate (3HB) units are the principal contributors to crystal formation, while the longer 3-hydroxyhexanoate (3HH) co-units are excluded from the ordered regions due to steric hindrance and conformational irregularity.^33,34^ More in detail, Sato et al.^43^ reported how these 3HH units remain confined to the amorphous phase, resulting in lower overall crystallinity and less densely packed lamellae compared to the homopolymer PHB. To gain insight into how GTH affects the crystalline structure of PHBH, WAXD analysis was conducted on the neat and plasticized formulations. Full diffractograms of all samples are shown in Figure 3A, while a magnified view focusing on the main peaks is reported in Figure 3B. All samples exhibited the typical reflections of the α-phase, with dominant peaks at 2θ of 13.5° and 16.9°, corresponding to the [020] and [110] planes, respectively. In addition, a third peak centered around 15.9°, assigned to the [011] plane and commonly attributed to the β-phase, was also observed (Figure S2).^44^ Although the addition of GTH did not significantly alter the position of the main diffraction peaks, indicating that the lattice parameters and *d*-spacing remained nearly constant, the relative intensities of the peaks changed considerably. As shown in Figure 3C, the intensities of the [020] and [110] peaks of the α-phase initially increased with GTH content, suggesting enhanced molecular ordering and crystal growth. At the same time, a substantial and progressive increase in the intensity of the [011] reflection of the β-phase was observed, suggesting a growing contribution from this phase. This trend is further illustrated in Figure 3D, which shows a clear increase in the relative intensity of the β-phase reflections compared with those of the α-phase. The presence of the β-phase was consistent with the DSC results, which showed a decrease in *T*_m_, consistent with the fact that the β-phase exhibits a lower thermal stability than the α-phase.^45^ The intensification of the β-phase signal, typically associated with extended zigzag chain conformations and looser packing, points to a structural reorganization of the crystalline domains. Although this polymorphic transformation is typically induced through mechanical stretching,^46–48^ its presence in these unstressed films suggests that internal molecular mechanisms are at play. As reported by Farrag et al.,^39^ the incorporation of plasticizers into PHBH did not result in any noticeable formation or intensification of β-phase peaks, indicating that the induction of such polymorphic transitions is not a general consequence of plasticizer addition, but rather a specific outcome related to the molecular characteristics and interactions of the additive used. It can be hypothesized that the intercalation of GTH between polymer chains increased the local free volume and perturbed the amorphous phase between α-lamellae, thus promoting conformational distortions that resembled the effects of mechanical deformation, as schematically illustrated in Figure 3E. In other words, the GTH molecules intercalation in the amorphous phase forced the α-lamellae apart, generating a localized molecular tensile stress similar to that induced by external tensile forces, that may trigger the formation of β structures from the α-phase, consistent with the observed increase in β[011] reflection intensity, and as also reported elsewhere.^48^ These findings also align with the free volume theory,^40^ for which the introduction of small plasticizer molecules, especially those with a branched structure such as GTH, not only lowers the system *T*_g_, but also contributes significantly to the creation of free volume, thereby enhancing chain mobility and promoting structural reorganization even in the absence of external force.

**Figure 3:**
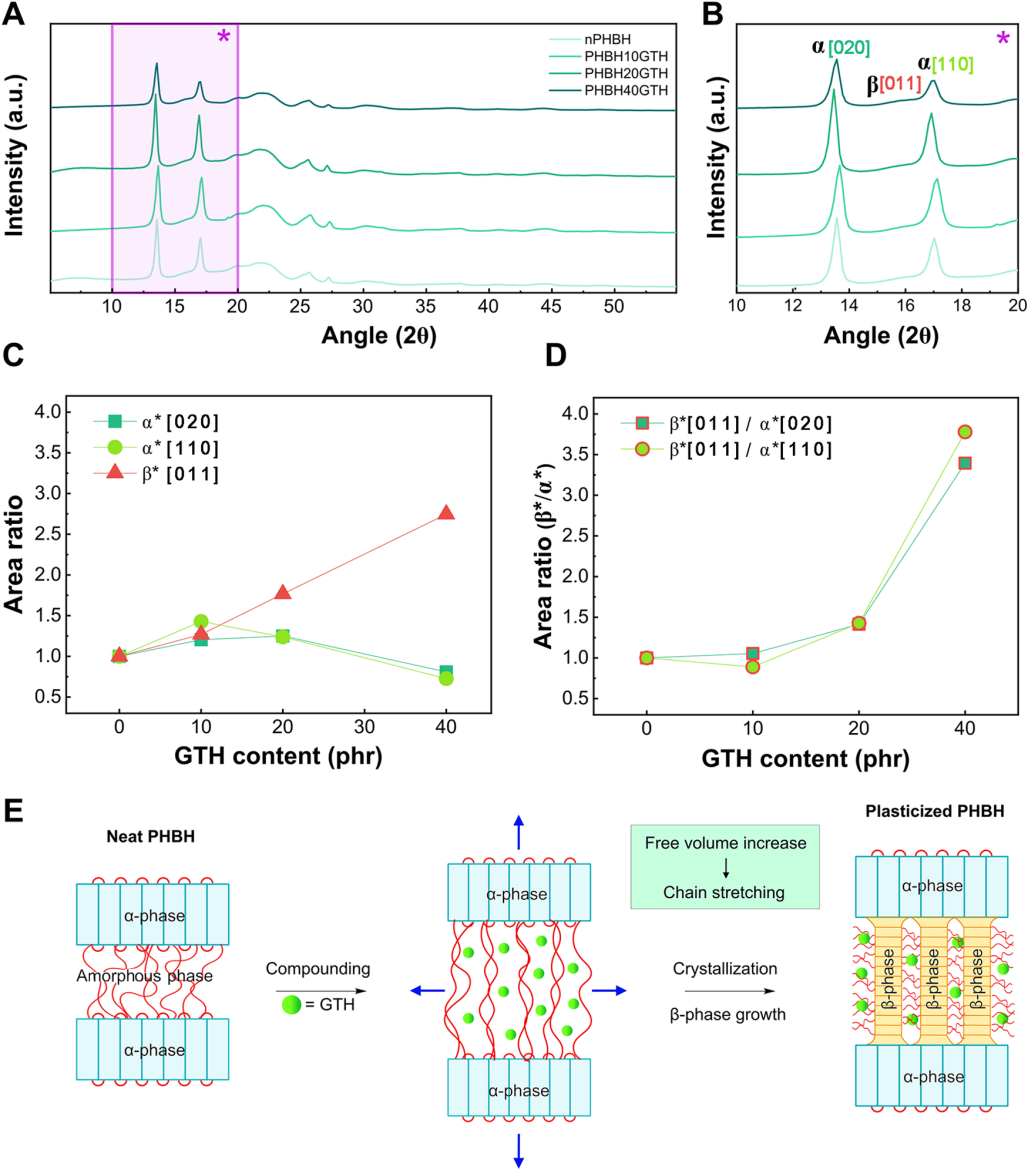
(A) Full WAXD diffractograms of neat and plasticized PHBH formulations. (B) Highlight of the 10–20° 2θ range of the WAXD diffractograms, focusing on the main reflection peaks: α [020],[110] and β-phase [011]. (C) Normalized peak area ratio *A*_phase(x)_^*^ for the α [020], α [110], and β [011] reflections, calculated with eq 2. (D) Relative intensity of β-phase reflection with respect to the α-phase reflections, obtained using eq 3. (E) Schematic illustration of GTH-induced free volume increase and molecular strain promoting the formation of β-phase structures in PHBH.

### 3.4 Mechanical Properties and Morphology

The mechanical performance of neat and plasticized films was assessed through uniaxial tensile tests, with results summarized in Table S2 and representative stress–strain curves shown in Figure S8A,B for PVC and PHBH compounds, respectively. Two key parameters, *E* and ε_break_, were used to evaluate the softening of the polymeric matrices induced by GTH. In the case of PVC, the neat formulation exhibited a high modulus of 1927 MPa and a limited elongation of 17%, reflecting its intrinsically rigid and amorphous nature. As shown in Figure 4A, GTH incorporation led to a sharp decrease in stiffness, with *E* values dropping to 853, 62, and 4 MPa for formulations containing 10, 20, and 40 phr, respectively. This softening was accompanied by a substantial improvement in ductility (Figure 4B), with ε_break_ reaching 656% at 20 phr. At 40 phr, a slight decrease in elongation (467%) was observed, potentially indicating a saturation of the plasticizing effect or early signs of phase separation at such a high plasticizer content. In the case of PHBH, the neat material initially exhibited the typical behavior of a semicrystalline biopolyester, with a relatively high *E* of 616 MPa and a very low ε_break_ of 5%, representative of its inherent brittleness. Upon incorporation of GTH, a clear plasticizing effect was observed, progressively softening the material and improved ductility (Figure 4C,4D). In fact, at a GTH content of 10 phr, the modulus dropped to 272 MPa, with a modest increase in ε_break_ to 8%. This softening effect became more pronounced at 20 phr, where *E* further decreased to 79 MPa and ε_break_ reached 18%, reflecting improved chain mobility and flexibility. The most significant change was observed at a GTH concentration of 40 phr, with *E* reduced to 74 MPa and ε_break_ reaching 38%, a remarkably high value for this inherently brittle material. This progressive evolution in mechanical behavior, although less dramatic than in PVC, still highlights the efficiency of GTH in plasticizing PHBH, an effect that can be attributed not only to the reduced friction between the polymeric chains induced by the plasticizer but also to the previously discussed β-phase formation observed with WAXD investigation. In fact, as reported by Yang et al.,^47^ the development of β-phase structures in PHA-based systems is shown to reduce rigidity and improve flexibility due to their less ordered and more flexible crystalline arrangement. SEM images (Figure 4E,F) show the morphological characteristics of the plasticized formulations and allow evaluation of the compatibility and dispersion of GTH within the studied polymers. In the PVC-based samples (Figure 4E), all formulations exhibited smooth and homogeneous cross-sectional surfaces with no visible evidence of phase separation. Surprisingly, even the formulation containing 40 phr of GTH, which exhibited a reduction in mechanical properties (Figure 4B), did not show any indication of a second phase formation. This was unexpected since the mechanical properties drop is often associated with plasticizer phase separation or poor polymer/plasticizer solubility.^15^ Conversely, the PHBH-based samples (Figure 4F) displayed more variation in the surface morphology depending on the GTH content. The neat PHBH formulation exhibited a distinct globular microstructure commonly attributed to crystalline spherulitic domains typical of semicrystalline PHAs. These globular features are associated with the radial growth of spherulites during the crystallization process, suggesting a relatively ordered crystalline arrangement. Interestingly, in PHBH10GTH and PHBH40GTH, this globular morphology appeared to be less defined or even suppressed. This may be attributed to the plasticizing effect of GTH, which, by increasing the chain mobility and free volume, interferes with the nucleation and growth of spherulites during solidification. At lower GTH content (10 phr), this may reflect an early stage interference with the crystallization process, while at higher concentrations (40 phr), the suppression might have been more pronounced due to increased molecular disorder and chain flexibility.

**Figure 4:**
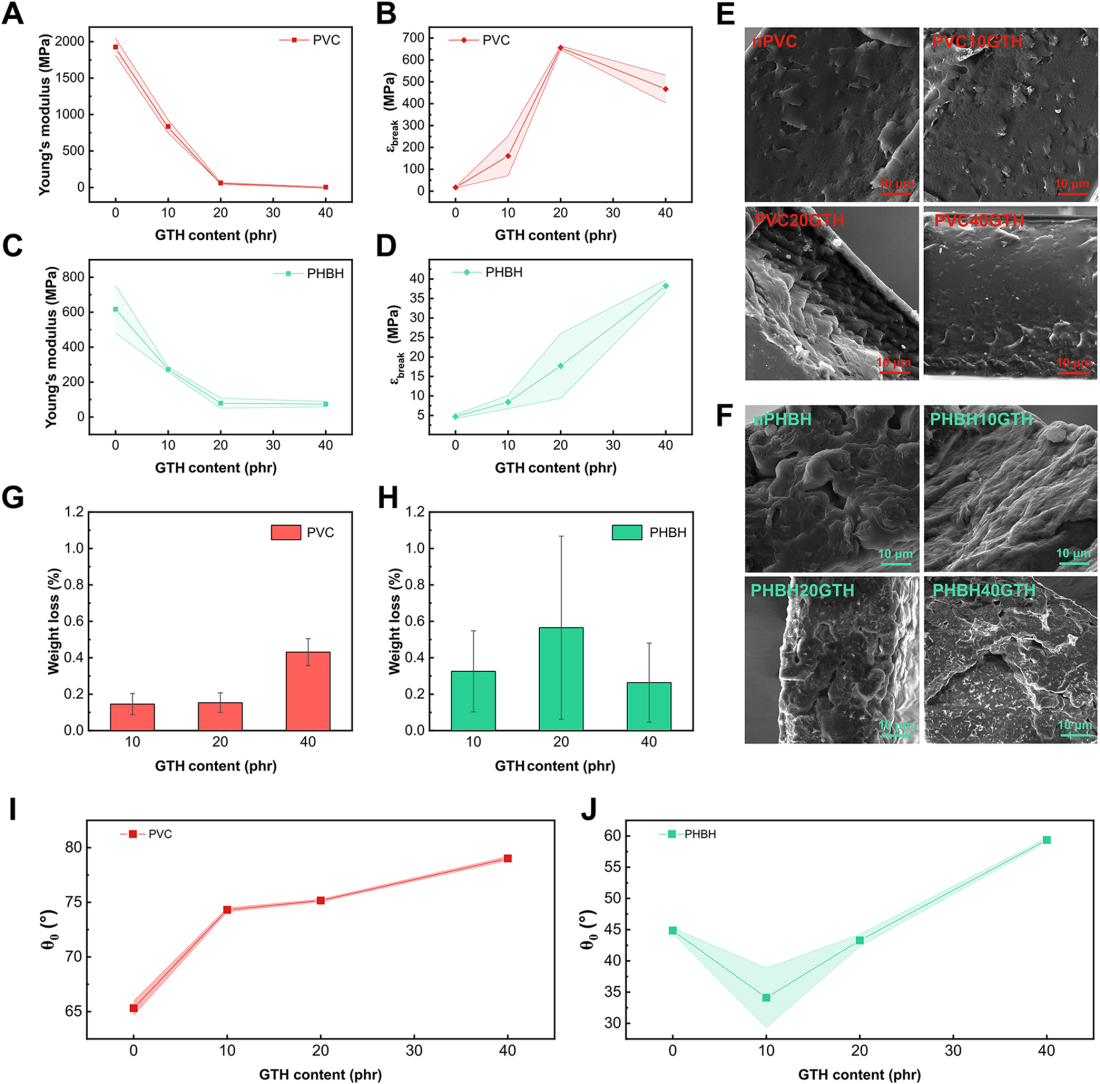
(A) Young’s modulus and (B) elongation at break (ε_break_) of PVC formulations with increasing GTH content. (C) Young’s modulus and (D) elongation at break (ε_break_) of PHBH formulations. Cross-sectional SEM micrographs of (E) PVC and (F) PHBH samples. Weight loss of (G) PVC and (H) PHBH plasticized compounds after 24 h immersion in water. Equilibrium water contact angle (θ_0_) of (I) PVC and (J) PHBH compounds, calculated by eq 5.

### 3.5 Migration Resistance and Surface Wettability

Plasticizer migration is a key factor in determining the long-term performance, durability, and environmental safety of plastic materials, while also providing insights about the compatibility between the polymer and the additive. Therefore, assessing the migration resistance of GTH is crucial to establish its suitability as a long-lasting and eco-friendly plasticizer. In order to evaluate GTH leachability, water-based migration tests were conducted by immersing plasticized PVC and PHBH films in water for 24 h, and the weight loss (calculated by eq 4) was measured as an indicator of plasticizer release. As shown in Figure 4G for PVC and Figure 4H for PHBH, all plasticized formulations exhibited very limited weight loss, with values remaining consistently below 0.6%, regardless of the plasticizer content. The minimal weight loss observed can be attributed to the good compatibility between GTH and the polymer chains, as supported by SEM imaging, where the absence of phase separation and the uniform dispersion of GTH likely prevented its leaching out of the films. These results suggest that GTH provides a stable and persistent plasticizing effect, even in aqueous environments, contributing to a prolonged material performance and reducing the risk of environmental contamination. The excellent retention of GTH within both PVC and PHBH matrices suggested not only favorable compatibility but also specific molecular interactions with the hosting polymers. According to the lubricity theory,^40^ an effective plasticizer typically contains both polar and nonpolar regions: the polar groups form interactions or anchor points with the polymer chains, while the nonpolar segments reduce intermolecular forces, facilitating chain mobility. In this context, GTH’s polar ester bonds can interact with the polar functionalities of both PVC and PHBH, while its nonpolar alkyl chains provide the lubricating effect observed by the modification of the thermal and mechanical properties of the plasticized formulations.

To complement the compounds’ characterization, the surface wettability of the plasticized films was investigated, as it provides insight on the material surface composition, polarity, and potential distribution of the plasticizer. The surface wettability of plasticized PVC and PHBH films was investigated using dynamic contact angle (CA) measurements performed via the Wilhelmy plate method,^49^ with water as the probe liquid. Advancing (θ_a_) and receding (θ_r_) angles were measured, and the equilibrium contact angle (θ_0_) was calculated by eq 5, through an energy-based geometric model that minimizes the effect of dynamic hysteresis.^37^ The calculated θ_0_ values and related standard deviations are summarized in Table S3 and plotted in Figure 4I,J for PVC and PHBH samples, respectively. For the PVC-based systems, a consistent and progressive increase in θ_0_ was observed with increasing GTH content, ranging from 65.3° for the neat polymer to 79.0° for the compound with 40 phr of the plasticizer (Figure 4I). This trend suggested a reduction in surface polarity and an enrichment of the hydrophobic component at the surface, consistent with the predominant aliphatic nature of the HA moieties. The smooth θ_0_ increase (Figure 4I) also supports the hypothesis of uniform distribution of GTH throughout the whole polymer and its likely surface reorientation during film formation. Importantly, no signs of phase separation were observed in SEM imaging (Figure 4E), indicating that this surface modification is not a result of plasticizer domains but rather an expression of thermodynamically favored molecular orientation or surface enrichment of GTH apolar segments. PHBH-based formulations displayed a more complex wettability profile. The neat PHBH film exhibited a relatively hydrophilic character, with a θ_0_ of 44.9°, in line with the typical polyester polarity. At low GTH concentrations (10 and 20 phr), θ_0_ values decreased slightly or remained relatively constant, suggesting good miscibility and homogeneous incorporation of the plasticizer within the polymer bulk. However, at 40 phr, a significant increase in θ_0_ was observed, reaching 59.4°, which indicates a more hydrophobic surface. This transition suggests that at high concentrations, GTH begins to accumulate or orient at the film–air interface, possibly due to saturation of favorable interactions within the polymer. However, the absence of visible phase separation in SEM images (Figure 4F) and the low weight loss in water migration tests (<0.6% even at 40 phr) suggest that any surface migration is limited to nanoscale rearrangements rather than to a macroscopic leaching.

## 4 Conclusions

This work presented glycerol trihexanoate (GTH) as a fully biobased plasticizer synthesized via a solvent-free esterification of glycerol and hexanoic acid, two renewable and nontoxic building blocks. The plasticizing performance of GTH was investigated in both a fossil-based amorphous polymer (PVC) and a semicrystalline biodegradable polyester (PHBH), enabling a comprehensive evaluation across two different polymeric systems. The plasticizing efficiency of GTH was confirmed through its impact on the thermal and mechanical properties of both polymers. In PVC, GTH reduced the *T*_g_ from 53 °C up to −3 °C at 40 phr, while in PHBH, with the same additive content, *T*_g_ decreased from 3 °C to −26 °C. GTH also demonstrated a clear ability to influence the crystalline organization of PHBH as, at increasing plasticizer content, the *T*_m_ decreased from 148 °C in the neat sample to 140 °C at 40 phr, while the degree of crystallinity progressively increased from 4 to 41%, indicating enhanced chain mobility and easier macromolecular reorganization into crystalline structures. Complementary WAXD analysis revealed a corresponding evolution in polymorphic composition, with a relative reduction in the α-phase intensity and a marked increase in the β-phase reflection. This evolution in the crystalline structure indicates that the presence of GTH within the polymer disrupted the regular packing of chains by increasing the local free volume. Such structural loosening likely introduced conformational strain at the molecular level, resembling the effect of mechanical stretching, thereby facilitating the formation of β-phase domains through alternative crystallization mechanisms. These structural rearrangements correlated with a significant improvement in the mechanical performance, particularly in terms of flexibility. The elongation at break increased dramatically in both polymer systems, rising from 17% to 656% in PVC with 20 phr of GTH, and from 5 to 38% in PHBH at 40 phr, confirming the very significant plasticizing effect imparted by GTH biobased plasticizer. SEM images revealed smooth and homogeneous fractured surfaces across all formulations with no observable phase separation, even at high plasticizer contents. Moreover, migration tests carried out in water showed negligible weight losses below 0.6% across all samples, indicating an excellent retention of the plasticizer within both polymer matrices. Complementary surface characterization through dynamic contact angle analysis showed a progressive increase in hydrophobicity with increasing GTH content for both PVC and PHBH films, suggesting partial surface orientation of the apolar chains of the plasticizer and offering further insight into the plasticizer–polymer interaction. These findings highlighted the potential of GTH as a sustainable alternative to conventional plasticizers. Thanks to its biobased origin, low leachability, and strong plasticizing effect, GTH might contribute to lowering the carbon footprint and toxicity of plasticized materials, supporting the transition to safer and more sustainable polymeric systems.

## Supplementary Material



## Data Availability

The authors confirm that the data supporting the findings of this study are available within the article and the Supporting Information. Additionally, raw data files are available from the corresponding author upon reasonable request.

## Notes

Pictures of the neat and plasticized formulations (Figure S1); WAXD spectra deconvolution and fitting curves (Figure S2); FTIR spectra of GLY and HA (Figure S3); representative TLC plate of the reaction mixture and main product (Figure S4); ^1^H NMR and ^13^C NMR spectra of purified GTH and related assignments (Figure S5); two-dimensional NMR analyses of purified GTH (Figure S6); summary of the thermal properties (Table S1); DSC thermograms of the PVC and PHBH formulations (Figure S7); summary of the mechanical properties (Table S2); representative stress–strain curves of the prepared formulations (Figure S8); and summary of the contact angles (Table S3) (PDF)
