# Supplementary material for: A Green Synthetic Approach to Glycerol Trihexanoate from Renewable Feedstocks for Enhanced Plasticization across Diverse Polymer Matrices
Source: ACS Appl Polym Mater. 2026 Mar 29;8(7):5258–69. doi: 10.1021/acsapm.6c00413 (PMC13078346; doi:10.1021/acsapm.6c00413)
Supplement: Supplementary file 1 [file ap6c00413_si_001.pdf]

SUPPORTING INFORMATION

A Green Synthetic Approach to Glycerol Trihexanoate from Renewable Feedstocks for Enhanced Plasticization Across Diverse Polymer Matrices

Luca Lenzi [a,b], Laura Martellosio [a,b], Marica Bianchi [b,c], Andrea Dorigato [b,c], Micaela Degli Esposti [a,b], Davide Morselli [a,b,*], Paola Fabbri [a,b]

[a] *Civil, Chemical, Environmental and Materials Engineering Department, University of Bologna, Via Terracini 28, 40131 Bologna, Italy*

[b] *National Interuniversity Consortium of Materials Science and Technology (INSTM), 50121 Firenze, Italy*

[c] *Department of Industrial Engineering, Università di Trento, Via Sommarive 9, 38123 Povo, Italy*

* Corresponding Author

Davide Morselli davide.morselli6@unibo.it

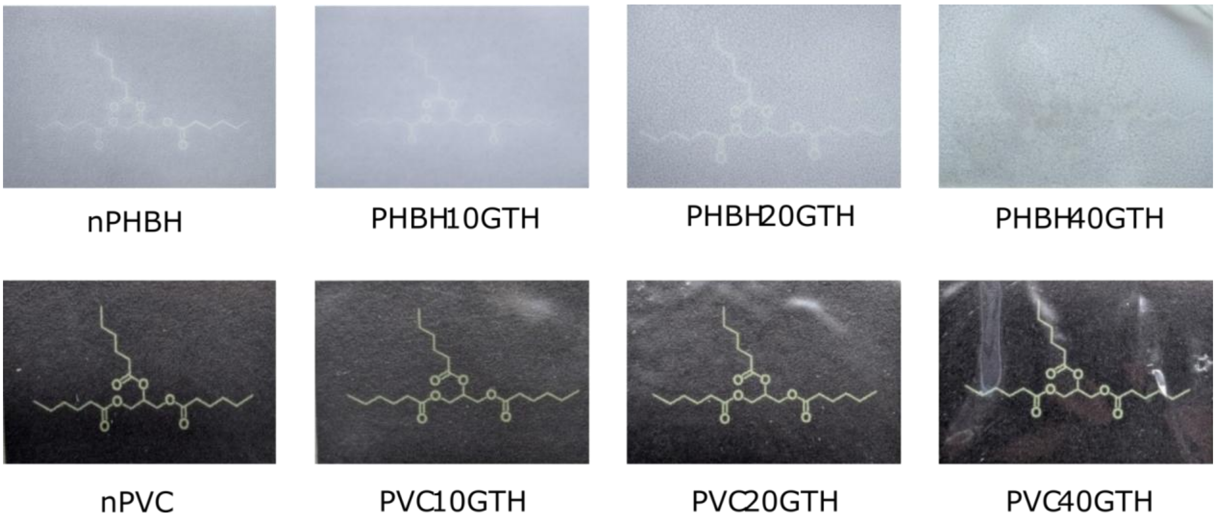

Figure S1. Photographs of the neat and plasticized PHBH and PVC films.

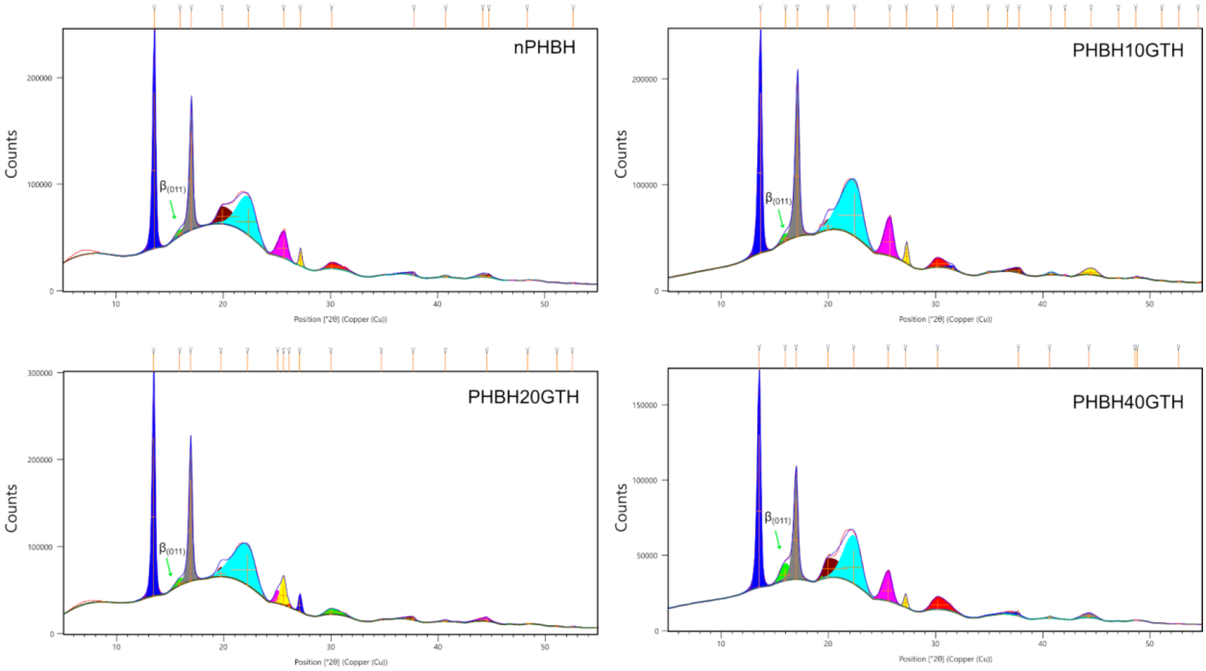

Figure S2. WAXD spectra deconvolution and fitting curves (in blue) of neat and plasticized PHBH samples.

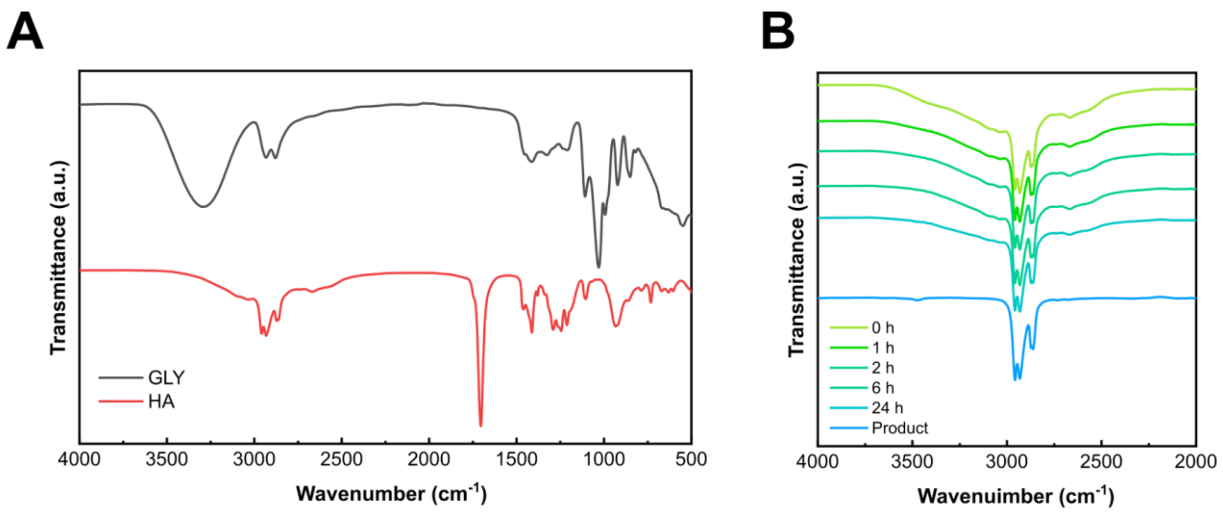

Figure S3. FTIR spectra of (A) GLY and HA. (B) inset on the broad hydroxyl stretching band (3000–3500 cm^{-1}) of the reaction mixture at different time points (green scale solid lines) and of purified GTH (light blue solid line).

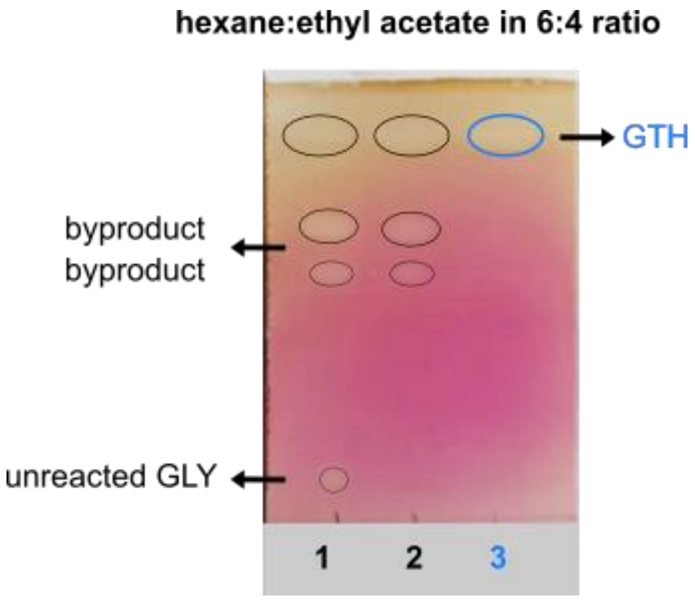

Figure S4. Representative TLC plate developed in KMnNO$_4$ and obtained using an eluent mixture of hexane:ethyl acetate (6:4 volume ratio). (1) is the reaction mixture at 24 hours, (2) is the product extracted with just ethyl acetate and (3) is the product extracted with the optimized procedure that is also reported in the Experimental Section.

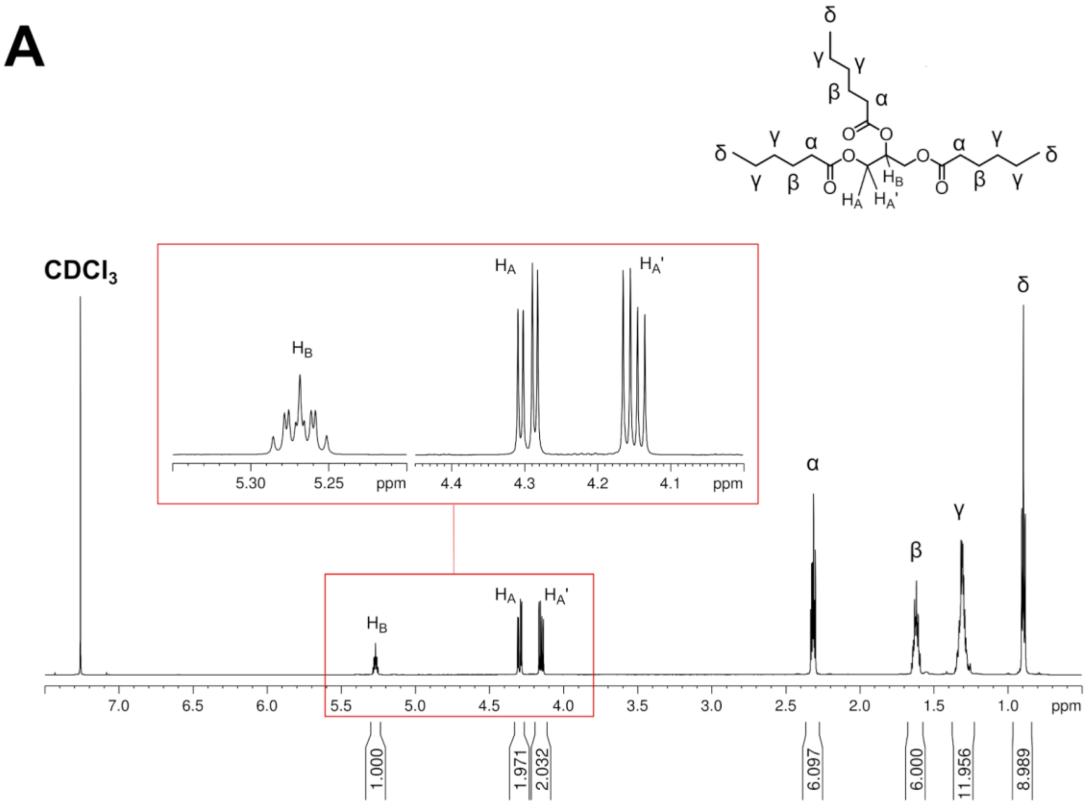

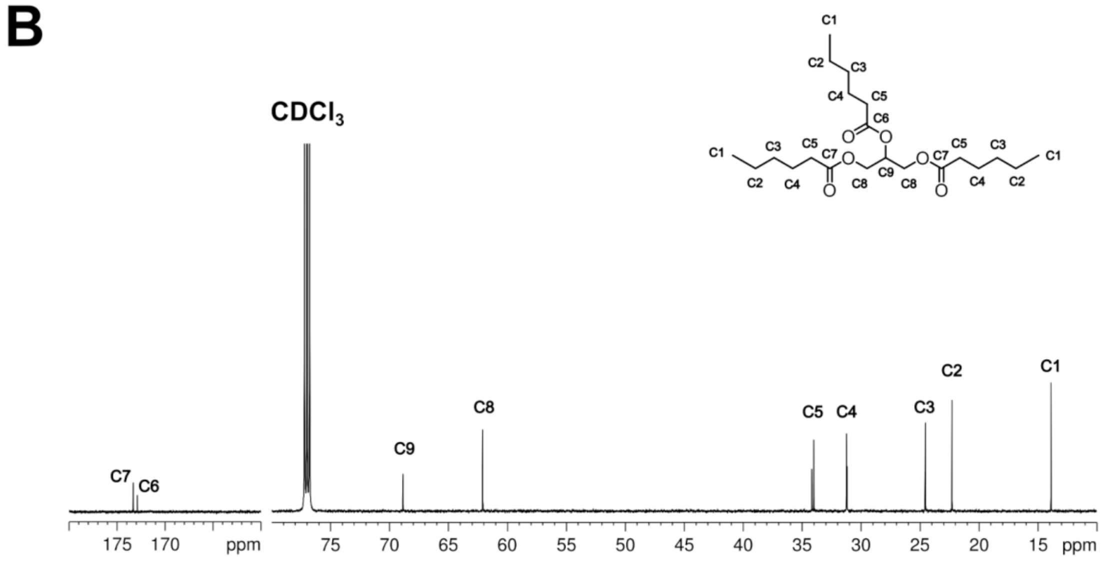

Figure S5. (A) ^{1}H-NMR spectrum of purified GTH. The inset shows the diastereotopic glycerol methylene protons. (B) ^{13}C-NMR spectrum of GTH.

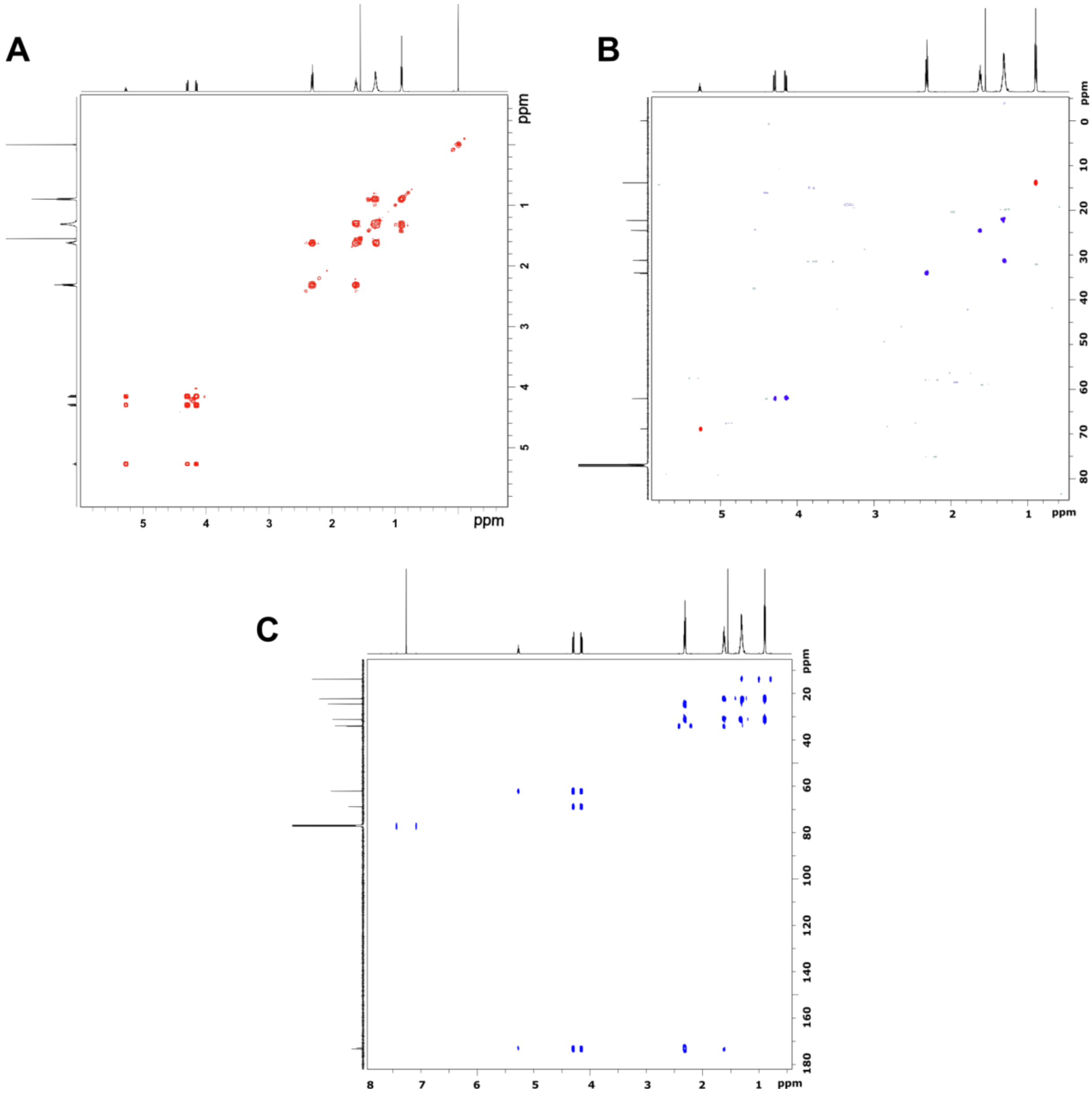

Figure S6. Two-dimensional NMR analyses of purified GTH, showing (A) COSY, (B) HSQC, and (C) HMBC spectra.

^{1}H- and ^{13}C-NMR assignments of the product

[$CH_3(CH_2)_4C(=O)O$]CH_2CH[$CH_3(CH_2)_4C(=O)O$]CH_2[$CH_3(CH_2)_4C(=O)O$]. The compound was obtained as a pale yellow oil (85% yield).

<u>^{1}H-NMR ($CDCl_3$, 400 MHz)</u> H_B δ = 5.27 (m, 1H, $CH_2\mathit{CH}CH_2$); H_A δ = 4.29 (dd, 2H, $C(=O)O\mathit{CH_2}CHCH_2$); $H_{A'}$ δ = 4.15 (dd, 2H, $C(=O)OCH_2CH\mathit{CH_2}$); *α* δ = 2.33 (t, 6H, $CH_3(CH_2)_3\mathit{CH_2}C(=O)O$); *β* δ = 1.63 (m, 6H, $CH_3(CH_2)_2\mathit{CH_2}CH_2C(=O)O$); *γ* δ = 1.35 (m, 12H, $CH_3\mathit{CH_2CH_2}CH_2CH_2C(=O)O$); *δ* δ = 0.89 (t, 9H, $\mathit{CH_3}CH_2CH_2CH_2CH_2C(=O)O$).

^{13}C-NMR ($CDCl_3$, 150 MHz) *C7, C6* δ = 173.1, 172.9 (*C*=O); *C9* δ = 69.9 (CH_2CHCH_2); *C8* δ = 62.1 (CH_2CHCH_2); *C5* δ = 34.2 ($CH_3CH_2CH_2CH_2CH_2C(=O)O$); *C4* δ = 31.6 ($CH_3CH_2CH_2CH_2CH_2C(=O)O$); *C3* δ = 24.4 ($CH_3CH_2CH_2CH_2CH_2C(=O)O$); *C2* δ = 22.5 ($CH_3CH_2CH_2CH_2CH_2C(=O)O$); *C1* δ = 13.9 ($CH_3CH_2CH_2CH_2CH_2C(=O)O$).

Table S1. Thermal properties, determined by DSC, of PVC and PHBH formulations that are reported as the polymer weight fraction (W_{pol}). Glass transition temperature (T_g), cold crystallization temperature (T_{cc}), cold crystallization enthalpy (ΔH_{cc}), melting temperature (T_m), melting enthalpy (ΔH_m) and crystallinity degree (X_c).

**Sample**	W_{pol}	T_g (°C)	T_{cc} (°C)	ΔH_{cc} (J·g^{-1})	T_m (°C)	ΔH_m (J·g^{-1})	X_c (%)
nPVC	1.0	53	-	-	-	-	-
PVC10GTH	0.9	37	-	-	-	-	-
PVC20GTH	0.8	9	-	-	-	-	-
PVC40GTH	0.6	-3	-	-	-	-	-
nPHBH	1.0	3	44	45	148	51	4
PHBH10GTH	0.9	-14	33	5	143	54	37
PHBH20GTH	0.8	-26	19	4	142	50	39
PHBH40GTH	0.6	-26	20	8	140	44	41

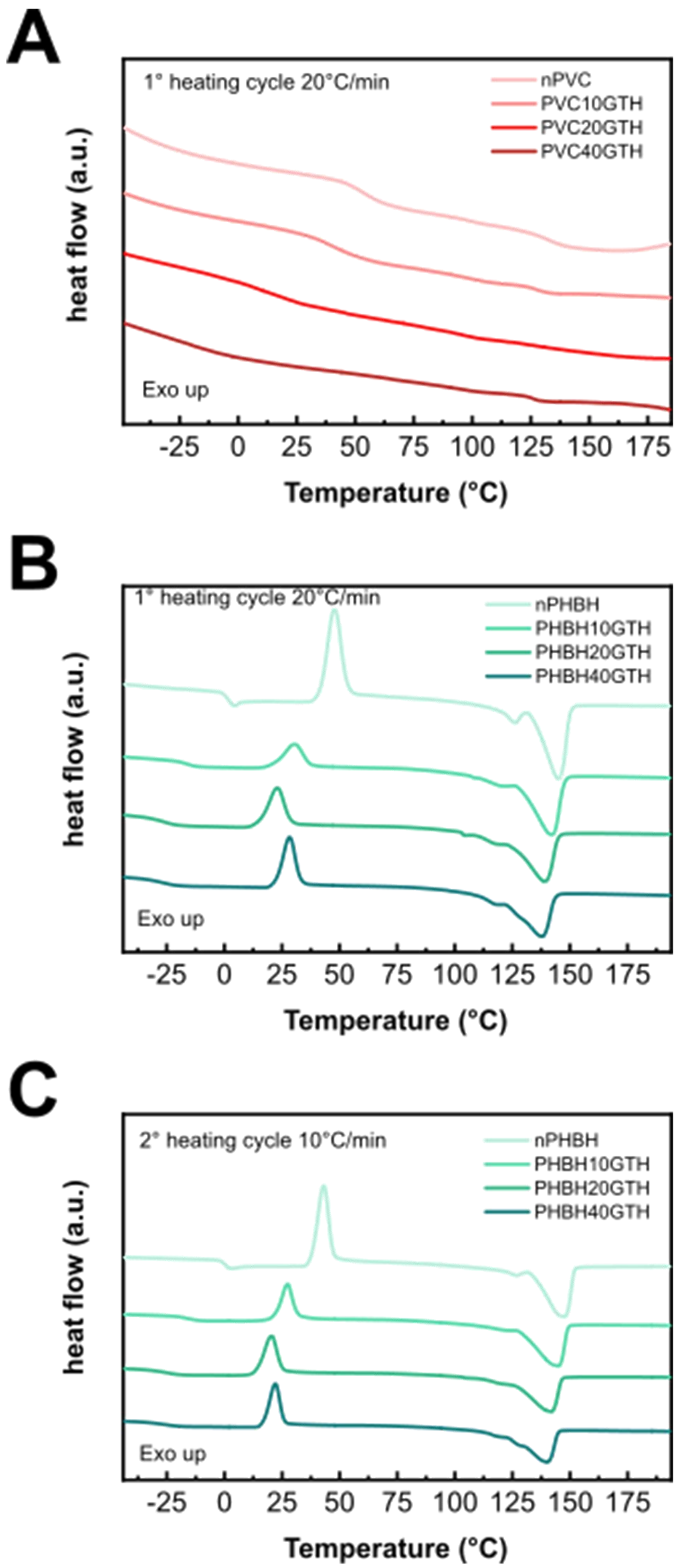

Figure S7. DSC thermograms of neat and plasticized PVC formulations. (A) First heating cycle at 20 °C·min^{-1}. (B) First heating cycle, at 20 ° C·min^{-1}, of PHBH samples. (C) Second heating cycle, at 10 °C·min^{-1}, of PHBH formulations.

Table S2. Young's modulus (E) and elongation at break (ε_{break}) evaluated by tensile tests performed on neat and plasticized samples. Values are reported as mean value ± standard deviation.

**Sample**	**Young's Modulus, E (MPa)**	**Elongation at break, ε_{break} (%)**
nPVC	1927 ± 117	17 ± 2
PVC10GTH	853 ± 89	161 ± 90
PVC20GTH	62 ± 5	656 ± 9
PVC40GTH	4 ± 1	467 ± 63
nPHBH	616 ± 136	5 ± 1
PHBH10GTH	272 ± 13	8 ± 2
PHBH20GTH	79 ± 28	18 ± 8
PHBH40GTH	74 ± 15	38 ± 2

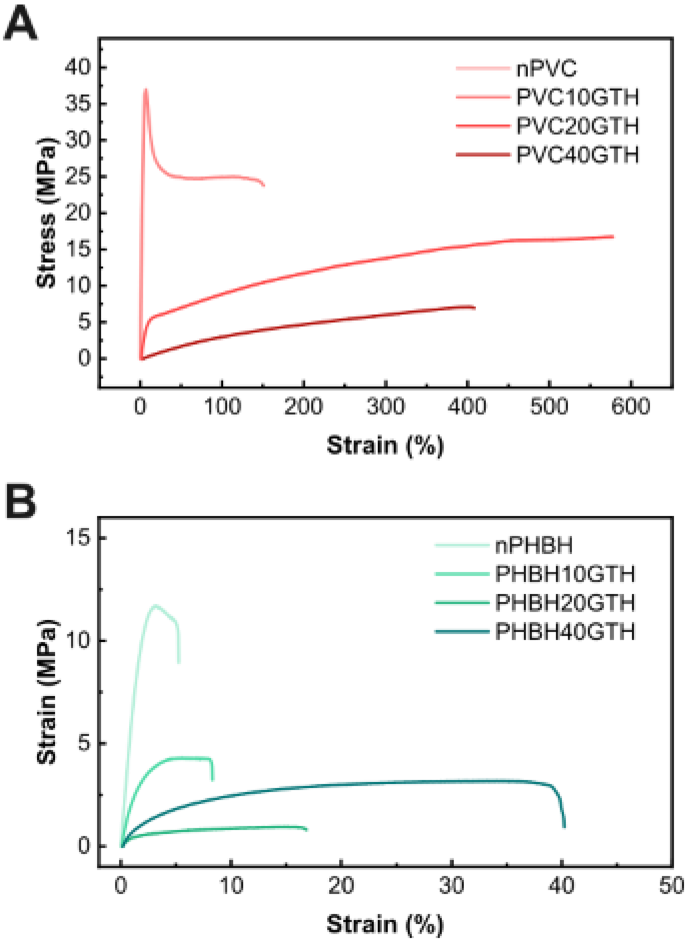

Figure S8. Representative stress–strain curves for (A) PVC and (B) PHBH formulations.

Table S3. Advancing contact angle (θ_a), receding contact angle (θ_r), and equilibrium contact angle (θ_0) of neat and plasticized PVC and PHBH formulations, with associated standard deviations.

**Polymer**	**GTH content (phr)**	**θ_a (°)**	**θ_r (°)**	**θ_0 (°)**
PVC	0	74.0 ± 1.1	57.2 ± 0.5	65.3 ± 0.6
	10	81.5 ± 0.3	67.7 ± 0.2	74.3 ± 0.4
	20	83.9 ± 0.1	67.2 ± 0.3	75.2 ± 0.3
	40	83.4 ± 0.3	74.9 ± 0.2	79.0 ± 0.2
PHBH	0	49.4 ± 0.4	40.4 ± 0.3	44.9 ± 0.4
	10	47.0 ± 3.1	20.7 ± 2.7	34.1 ± 2.8
	20	48.7 ± 1.1	37.9 ± 0.7	43.3 ± 0.8
	40	68.5 ± 0.4	50.7 ± 0.3	59.4 ± 0.4
